# A Comprehensive Evaluation of the Association between Polymorphisms in* XRCC1*,* ERCC2*, and* XRCC3* and Prognosis in Hepatocellular Carcinoma: A Meta-Analysis

**DOI:** 10.1155/2019/2408946

**Published:** 2019-06-12

**Authors:** Yan Zhao, Erjiang Zhao, Junhui Zhang, Yuanyuan Chen, Junli Ma, Hailiang Li

**Affiliations:** ^1^Department of Minimally Invasive Interventional Radiology, Affiliated Tumor Hospital of Zhengzhou University, Zhengzhou 450003, China; ^2^Department of Biostatistics, Affiliated Tumor Hospital of Zhengzhou University, Zhengzhou 450003, China; ^3^Henan Provincial Key Lab For Control of Coronary Heart Disease, Fuwai Central China Cardiovascular Hospital, Zhengzhou, China; ^4^Collage of Nursing, HeNan University, Kaifeng 475000, China

## Abstract

**Purpose:**

Associations between* XRCC1*,* XRCC3*, and* ERCC2* gene polymorphism and prognosis have been investigated in several cancers. The aim of this meta-analysis was to assess the prognostic value of* XRCC1*,* XRCC3*, and* ERCC2* gene polymorphism in hepatocellular carcinoma (HCC).

**Methods:**

A systematic literature search was performed to identify relevant studies in PubMed, Embase, and the Cochrane library up to December 2018. The prognostic values of* XRCC1*,* XRCC3*, and* ERCC2* polymorphisms in HCC were estimated using crude HRs with 95% CIs.

**Results:**

Ten studies involving 2687 patients were included in the quantitative analysis. There were no statistically significant associations between* XRCC1* rs1799782 C>T,* XRCC1* rs25487 G>A, and* ERCC2* rs1799793 G>A polymorphisms and overall survival (OS). OS was significantly longer for the* ERCC2* rs13181 CC genotype than for AA (CC vs. AA: HR = 0.33, 95% CI = 0.15–0.72). A significantly lower OS was observed for patients with the CT genotype compared with the CC genotype at* XRCC3* rs861539 (CT vs. CC: HR = 1.64, 95% CI = 1.11–2.42).

**Conclusion:**

The* ERCC2* rs13181 A>C polymorphism and* XRCC3* rs861539 C>T polymorphism may be predictive markers for prognosis in patients with HCC. Well-designed studies with larger sample sizes are needed to verify our findings.

## 1. Introduction

Liver cancer is one of the most common malignancies. It was the sixth most commonly diagnosed cancer and the fourth cause of cancer-related death worldwide in 2018 and accounts for approximately 841,000 new cases and 782,000 deaths annually [1]. Hepatocellular carcinoma (HCC) is the most common primary cancer of the liver, accounting for 75–85% of liver cancer cases. According to the stage of HCC, patients receive various treatments, ranging from surgery to radiotherapy, transarterial chemoembolization (TACE), and targeted therapy [2]. Although treatment advances have improved clinical outcomes in the last few decades, the 5-year survival rate has remained at only about 18% from 2005 to 2011 [1].

The development of HCC is a multifactorial process involving an insidious onset, rapid progression, and high mortality rates. The fundamental pathogenic event in carcinogenesis is the accumulation of DNA damage and errors in DNA repair. In response to DNA damage, specific DNA repair mechanisms are activated. The major DNA repair pathways are mismatch repair (MMR), base excision repair (BER), nucleotide excision repair (NER), and double-strand break repair (DSBR) [3]. Polymorphisms in genes involved in DNA repair are likely to play an important role in the prognosis of HCC and are useful factors for determining the risk of cancer progression or recurrence. Various genetic factors are predicted to affect treatment efficiency and prognosis in patients with HCC, such as X-ray cross-complementing group 1 (*XRCC1*), X-ray repair cross-complementing group 3 (*XRCC3*), and excision repair cross-complementation group 2 (*ERCC2*). Many previous studies have investigated the associations between polymorphisms in these genes and prognosis [4–7]. In 2012, Jung et al. [8] found that* XRCC1* rs25487,* ERCC5* rs2018836,* ERCC5* rs3818356, and* XRCC4* rs1805377 have significant effects on survival. Another study suggested that* ERCC2*-312 genotypes but not* XRCC1*-194 were independent risk factors for poor prognosis in HCC [9]. Han et al. [10] showed that the* XRCC1* Gln allele and* XRCC3* T allele are related to a poor prognosis in HCC.

Despite a number of recent studies of the relationships between* XRCC1*,* XRCC3*, and* ERCC2* gene polymorphisms and prognosis in HCC [8–16], the results are inconclusive. We performed a meta-analysis to comprehensively assess the correlation between these polymorphisms and prognosis in HCC.

## 2. Materials and Methods

### 2.1. Search Strategy

The present study was conducted according to the PRISMA guidelines for systematic reviews and meta-analyses [17]. A systematic literature search of PubMed, Embase, and the Cochrane library was conducted to identify studies published up to December 2018. The following retrieval strategy was used in accordance with ICPO (liver neoplasms OR hepatocellular cancer OR hepatocellular carcinoma OR liver cancer OR liver carcinoma OR hepatocellular neoplasms) AND (*XRCC1* OR X-ray repair cross-complementing group1 OR* XRCC3* OR X-ray repair cross-complementing group 3 OR* ERCC1* OR excision repair cross-complementation group 1 OR* ERCC2* OR excision repair cross-complementing group 2 OR* XPD* OR xeroderma pigmentosum group D) AND (prognosis OR survival). The reference lists of the identified studies were examined to identify additional studies. When multiple studies evaluated the same population, the most recent and the largest study was included.

### 2.2. Inclusion and Exclusion Criteria

The following inclusion criteria were applied. (1) Patients in the study were diagnosed with HCC. (2) Genotype frequencies could be extracted for at least one of the five polymorphisms. (3) Studies provided sufficient data for the prognostic effects in patients with HCC. The following exclusion criteria were applied: (1) studies with repeated data, (2) studies lacking sufficient data, and (3) studies of patients with carcinomas other than the liver.

### 2.3. Data Extraction and Quality Assessment

Two investigators extracted data independently, including information on authors, year, country, ethnicity, number of patients, SNP sites, quality assessment scores, and HRs with 95% confidence intervals (CIs). If the HR and 95% CI could not be obtained directly, they were extracted using the methods of Parmar [18], Tierney [19], and Williamson [20]. Discrepancies were resolved by consensus. The Newcastle-Ottawa scale (NOS) was used to evaluate the quality of the identified studies [21]. The NOS includes 3 categories, selection (0–4 points), comparability (0–2 points), and outcome (0–3 points) [22]. The total scores ranged from 0 to 9. NOS scores of 0–3, 4–6, and 7–9 were considered low, moderate, and high.

### 2.4. Statistical Analysis

The strength of the associations between five gene polymorphisms and overall survival (OS) in HCC was estimated using crude HRs with 95% CIs. The Chi squared-based Q-test and *I*^2^ statistic were utilized to evaluate heterogeneity. When P < 0.10 or *I*^2^ > 25% for the Q-test, a random effects model (DerSimonian and Laird method) was used to evaluate the pooled HR. Otherwise, a fixed-effects model (Mantel-Haenszel method) was used [23]. In addition, a sensitivity analysis was used to estimate the stability of our results by omitting one study at a time and recalculating the pooled HR. Funnel plots and Egger's test were used to assess publication bias [24]. All statistical analyses were implemented using STATA (version 12.0; STATA Corporation, College Station, TX, USA) and two-sided P-values were obtained.

## 3. Results

### 3.1. Literature Search

**Figure 1:**
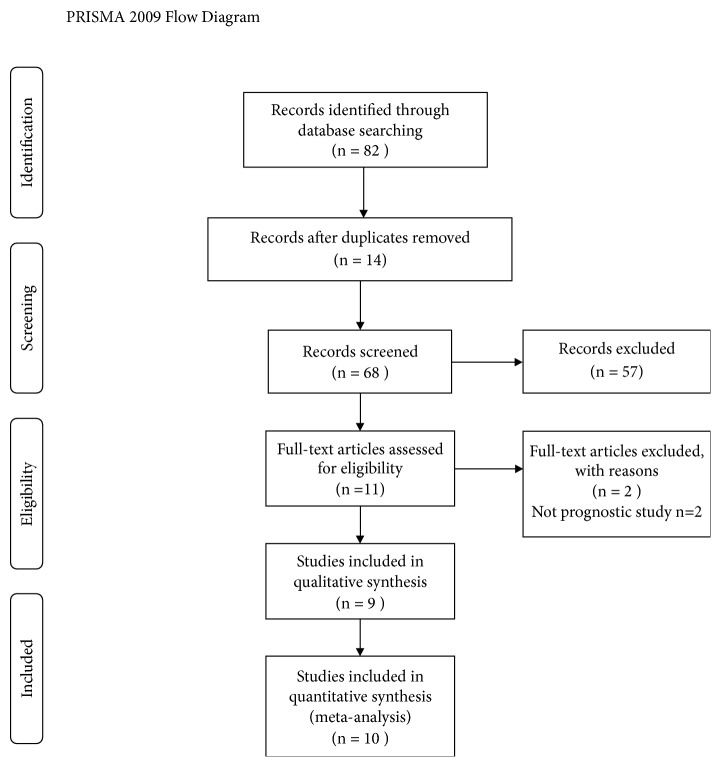
The flow chart of included studies in this meta-analysis.

The study search and selection strategies are presented in Figure 1. In total, 82 potentially relevant studies were retrieved from PubMed, Embase, and the Cochrane library. Ten duplicate studies were excluded and 57 studies were excluded after reading the titles and abstracts. After further review of the full-length texts, 2 studies did not examine prognosis and were excluded. Finally, we identified 9 eligible articles including 10 studies for inclusion in the meta-analysis.

### 3.2. Characteristics of Eligible Studies

The characteristics of studies included in the meta-analysis are shown in Table 2. There were 8 studies of Asian populations and 2 studies of Caucasians. The sample sizes of eligible studies ranged from 50 to 708. Five studies reported an association between the* XRCC1* rs25487 G>A polymorphism and prognosis in HCC, 3 studies reported an association for* XRCC1* rs1799782 C>T, 2 studies reported an association for* ERCC2* rs13181 A>C, 3 studies reported an association for* ERCC2* rs1799793 G>A, and 2 studies reported an association for* XRCC3* rs861539 C>T (Table 1). The NOS scores ranged from 5 and 9, demonstrating that the quality of the eligible studies was acceptable.

### 3.3. Quantitative Synthesis

**Figure 2:**
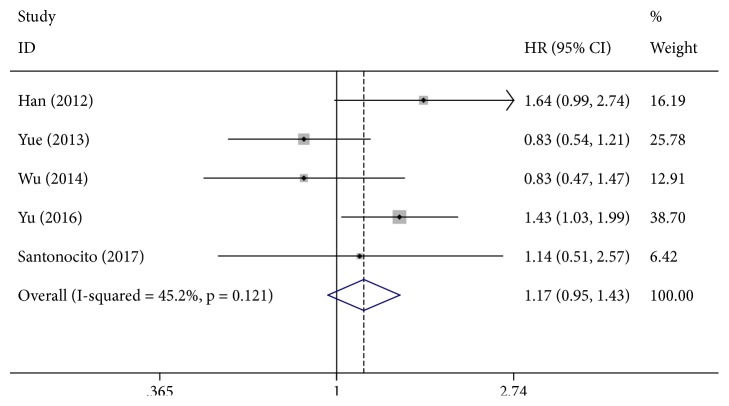
Forest plot for the association between XRCC1 rs25487 and overall survival for HCC patients (GA VS. GG).

Eight studies involving 2465 patients were included in the final analysis of the relationship between the* XRCC1* rs25487 G>A polymorphism and OS in HCC (Table 3). Our meta-analysis showed that there were no statistically significant associations between the* XRCC1* rs25487 G>A polymorphism and the OS (AA vs. GG: HR = 0.97, 95% CI = 0.51–1.87; GA vs. GG: HR = 1.17, 95% CI = 0.95–1.43, Figure 2; AA+GA vs. GG: HR = 1.25, 95% CI = 0.83–1.88).

Three studies with a total of 621 patients with advanced HCC were eligible for analyses of the association between the* XRCC1* rs1799782 C>T polymorphism and the OS. No significant association was detected between* XRCC1* rs1799782 C>T and prognosis in HCC (TT vs. CC: HR = 0.72, 95% CI = 0.48–1.08; CT vs. CC: HR = 0.88, 95% CI = 0.63–1.22).

**Figure 3:**
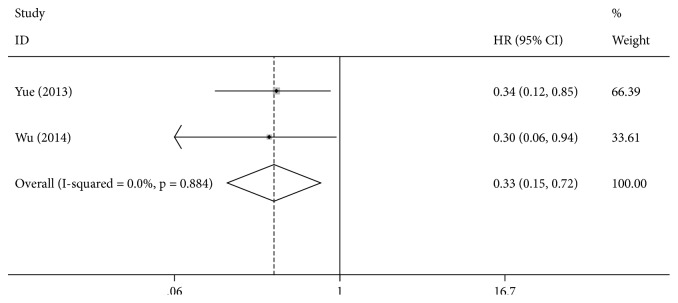
Forest plot for the association between ERCC2 rs13181 and overall survival for HCC patients (CC VS.AA).

Two eligible studies were identified for the analysis of the correlation between the* ERCC2* rs13181 A>C polymorphism and the OS.* ERCC2* rs13181 CC genotype carriers had a significantly longer OS than that of AA genotype carriers (CC vs. AA: HR = 0.33, 95% CI = 0.15–0.72, Figure 3), but there was no significant difference between the CC genotype and the AA genotype (CA vs. AA: HR =0.83, 95% CI = 0.62–1.12).

Three studies were applicable for analyzing the association between the* ERCC2* rs1799793 G>A polymorphism and the OS. The pooled results showed no significant association for any genetic model (AA vs. GG: HR = 0.74, 95% CI = 0.49–1.11; GA vs. GG: HR = 0.93, 95% CI = 0.67–1.28).

**Figure 4:**
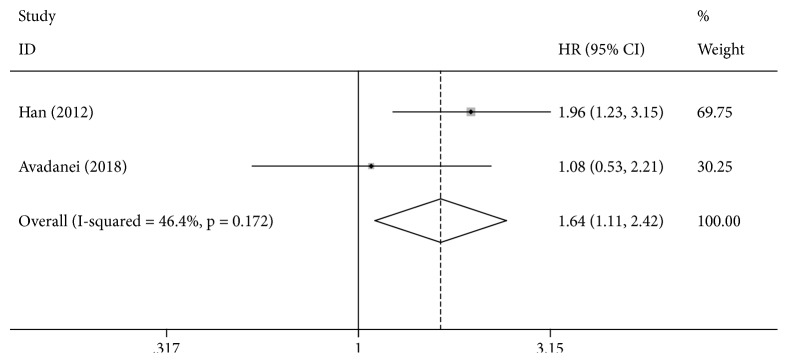
Forest plot for the association between XRCC3 rs861539 and overall survival for HCC patients (CT VS.CC).

Data from 2 studies were eligible for the analysis of the association between the* XRCC3* rs861539 C>T polymorphism and the OS. A significantly lower OS was observed for the CT genotype compared with the CC genotype (CT vs. CC: HR = 1.64, 95% CI = 1.11–2.42, Figure 4).

### 3.4. Publication Bias and Sensitivity Analysis

**Figure 5:**
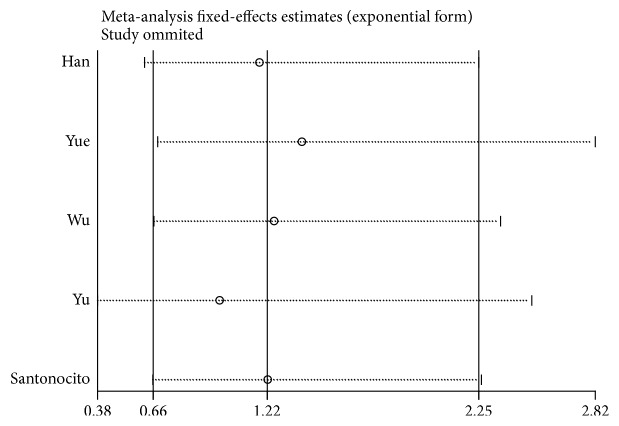
Sensitivity analysis between xrcc1 rs25487 and overall survival for HCC patients (GA VS.GG).

**Figure 6:**
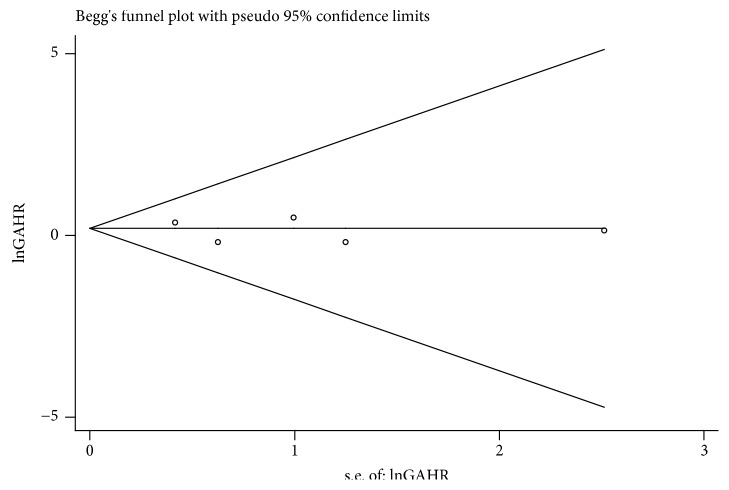
Funnel plot between xrcc1 rs25487 and overall survival for HCC patients (GA VS.GG).

Funnel plots and Egger's tests were used to assess publication bias. The funnel plot did not show apparent asymmetry in the overall population. Egger's test demonstrated funnel plot symmetry for the* XRCC1* rs25487 G>A polymorphism (AA vs. GG, P = 0.585; GA vs. GG, P = 0.653, Figure 5; AA+GA vs. GG, P = 0.802). In addition, we performed a sensitivity analysis to estimate the stability of our results by omitting one study at a time and recalculating the pooled HR. No single study changed the corresponding pooled HR and 95% CI (Figure 5), indicating that the results of our meta-analysis were statistically robust (Figure 6).

## 4. Discussion

DNA repair systems play a fundamental role in maintaining the integrity of genomic DNA. Polymorphisms in genes related to DNA repair mechanisms, including BER (e.g.,* XRCC1*), NER (e.g.,* ERCC2*), and DSBR (e.g.,* XRCC3*), may be instrumental in carcinogenesis, drug responses, treatment efficiency, and survival in HCC, thus affecting prognosis.

*XRCC1* is located on chromosome 19q13.2 and belongs to the BER pathway. It constantly repairs DNA base lesions and single-strand breaks caused by endogenous and exogenous mutagens; it has a central role in the BER pathway by interacting with other DNA repair proteins [25]. More than 300 single nucleotide polymorphisms have been identified in* XRCC1*. Among them, the* XRCC1* rs25487 G>A and rs1799782 C>T polymorphisms are the most well studied. Variation in* XRCC1* expression may modulate cancer sensitivity, clinical treatment efficiency, and prognosis. However, in our study, we found that neither* XRCC1* rs25487 G>A nor rs1799782 C>T influenced prognosis in HCC. A sensitivity analysis indicated that the results were statistically robust. Heterogeneity was detected in the AA vs. GG and AA+GA vs. GG model for the* XRCC1* rs25487 G>A polymorphism, indicating variability. Heterogeneity may have been caused by different study characteristics, such as ethnicity, HBV status, sample size, or cure method. The random effects model was used to determine the pooled HR. Because only 1 in 5 studies focused on Caucasians and data for African populations were not available, studies of different ethnicities are needed to further evaluate this locus.

NER is a powerful and complicated DNA damage removal pathway. The* ERCC2* enzyme, a critical element in NER, contributes to DNA repair by participating in DNA unwinding and structurally identifying DNA lesions, including large adducts and thymidine dimmers. rs13181 A>C and rs1799793 G>A are important polymorphisms in the* ERCC2* gene. Previous research has shown that the wild-type genotype AA is associated with a higher DNA repair capacity as compared to that of the CC genotype in the rs13181 polymorphism [26]. Qian et al. suggested that the* ERCC2* rs13181 A>C polymorphism has prognostic value in patients with colorectal cancer undergoing oxaliplatin-based chemotherapy [27]. A meta-analysis demonstrated that the rs13181 A>C and rs1799793 G>A polymorphisms are significantly correlated with the response to chemotherapy for patients with osteosarcoma [28]. Considering the importance of the NER pathway in tumors, polymorphisms in* ERCC2* were expected to have an influence on prognosis, but this was only assessed in two studies. Although we did not find that the* ERCC2* rs1799793 G>A polymorphism is associated with OS, our meta-analysis demonstrated that patients with the* ERCC2 *rs13181 CC genotype have significantly longer OS than that of AA genotype carriers. Further studies of the association between* ERCC2* polymorphisms and response to chemotherapy for patients with HCC are needed.

The* XRCC3* protein, a member of the DSBR pathway, plays an important role in homologous recombination and is therefore critical for chromosomal integrity and the stability of the genome. In 2012, Han et al. [10] found that individuals with* XRCC3* CT (HR = 1.96, 95% CI = 1.23–3.15) and TT genotypes (HR = 2.98, 95% CI = 1.77–7.54) have a significantly higher risk of HCC than that of individuals with the* XRCC3* CC genotype. In addition, Avadanei and collaborators [16] evaluated the rs861539 C>T polymorphism and observed a better survival only for the homozygote genotype (TT) compared to the heterozygote genotype (CT) but no differences between heterozygote TC and wild-type CC genotypes. We also performed a comprehensive evaluation of the* XRCC3* rs861539 C>T polymorphism and OS. We found a significantly lower OS for the CT genotype than the CC genotype. We suspected that the* XRCC3* rs861539 C>T polymorphism is a promising marker for prognosis in HCC. However, we did not perform a systematic literature review to comprehensively evaluate the effects of* XRCC3* TT and TC genotypes on prognosis in HCC owing to the lack of data. The relation between* XRCC3* and prognosis in HCC should be evaluated in future work. Because only two eligible studies were included in the meta-analysis, our results should be interpreted with caution. Studies with larger sample sizes are needed to obtain more definitive conclusions.

Several limitations of our study should be addressed. First, the analyses of* ERCC2* and* XRCC3* polymorphisms were based on only a few eligible studies. Second, the results of our comprehensive analyses were based on unadjusted estimates owing to the lack of adjusted data. Third, ethnicity, therapeutic regimen, and HBV infection status are very important factors for stratified analyses. However, few studies provided sufficient data for subgroups, making such stratified analyses impossible.

## 5. Conclusions

In conclusion, the* ERCC2 rs13181 A>C* polymorphism and* XRCC3 rs861539 C>T *polymorphisms are potential prognostic markers in HCC. The* ERCC2* rs13181 CC genotype was associated with a significantly longer OS than that of the AA genotype. The carriers of the CT genotype had a poor prognosis compared with that of the CC genotype carriers. Well-designed studies with larger sample sizes are needed to verify our findings.

## Figures and Tables

**Table 1:** Polymorphism involved in this study.

Genes	Polymorphisms	NCBI SNP ID	Allele	genotypes	References
XRCC1	G28152A (Arg399Gln)	rs25487	G^a^	GG	[8, 10–15]
				GA	
			A^b^	AA	
	C26304T (Arg194Trp)	rs1799782	C	CC	[9, 11, 12]
				CT	
			T	TT	
ERCC2	G934A (Asp312Asn)	rs1799793	G	GG	[9, 11–13]
				GA	
			A	AA	
	A2251C (Lys751Gln)	rs13181	A	AA	[9, 11, 12]
				AC	
			C	CC	
XRCC3	C722T (Thr241Met)	rs861539	C	CC	[10, 16]
				CT	
			T	TT	

^a^wild allele; ^b^mutant allele.

**Table 2:** Characteristics of studies included in this meta-analysis.

Study	Year	Country	Ethnicity	Number of patients	Age	HBV	SNP loci	scores of quality evaluation
Jung1 [8]	2012	Korea	Asian	708	53.3 ± 8.3	+	xrcc1 rs25487	8
Jung2 [8]	2012	Korea	Asian	314	53.3 ± 8.3	+	xrcc1 rs25487	8
Han [10]	2012	China	Asian	112	50.8±8.5	mixed	xrcc1 rs25487, xrcc3 rs861539	7
Yue [11]	2013	China	Asian	231	50.9±9.6	mixed	xrcc1 rs25487, xrcc1 rs1799782, ercc2 rs13181, ercc2 rs1799793	7
Wu [12]	2014	China	Asian	218	52.2 ± 8.5	+	xrcc1 rs25487, xrcc1 rs1799782, ercc2 rs13181, ercc2 rs1799793	6
Wang [13]	2016	China	Asian	308	53( 25–80)	Mixed	xrcc1 rs25487,ercc2 rs13181	7
Yu [14]	2016	China	Asian	485	⩽60,418; >60,67	+	xrcc1 rs25487	7
Santonocito [15]	2017	Italy	Caucasian	89	66.3±10.5	mixed	xrcc1 rs25487	6
Guan [9]	2017	China	Asian	172	50.4±4.8	Not reported	xrcc1 rs1799782, ercc2 rs1799793	6
Avadanei [16]	2018	romania	Caucasian	50	⩽65,28; >65,22	mixed	xrcc3 rs861539	5

**Table 3:** Meta-analysis of the association between XRCC1, ERCC2, and XRCC3 and overall survival for HCC patients.

Genetic comparisons	No. of studies	Test of association	Model	Test of heterogeneity	
		HR(95% CI)		P	I^2^(%)
xrcc1 rs25487					
AA vs. GG	5	0.97(0.51-1.87)	R	0.001	82.2
GA vs. GG	5	1.17(0.95-1.43)	F	0.121	45.2
AA+GA vs. GG	5	1.25(0.83-1.88)	R	0.002	76.3
xrcc1 rs1799782					
TT vs. CC	3	0.72(0.48-1.08)	F	0.849	0
CT vs. CC	3	0.88(0.63-1.22)	F	0.853	0
ERCC2 rs13181					
CC vs. AA	2	0.33(0.15-0.72)	F	0.884	0
CA vs. AA	2	0.83(0.62-1.12)	F	0.971	0
ERCC2 rs1799793					
AA vs. GG	3	0.74(0.49-1.11)	F	0.840	0
GA vs. GG	3	0.93(0.67-1.28)	F	0.780	0
XRCC3 rs861539					
CT vs. CC	2	1.64(1.11-2.42)	F	46.4	12

HR: hazard ratio; CI: confidence interval; vs: versus; F: fixed effect model; R: random effect model.
